# Exploiting repetitive sequences and BAC clones in *Festuca pratensis* karyotyping

**DOI:** 10.1371/journal.pone.0179043

**Published:** 2017-06-07

**Authors:** Joanna Majka, Tomasz Książczyk, Agnieszka Kiełbowicz-Matuk, David Kopecký, Arkadiusz Kosmala

**Affiliations:** 1 Institute of Plant Genetics, Polish Academy of Sciences, Poznań, Poland; 2 Institute of Experimental Botany, Centre of the Region Haná for Biotechnological and Agricultural Research, Olomouc, Czech Republic; Universite Laval, CANADA

## Abstract

The *Festuca* genus is thought to be the most numerous genus of the Poaceae family. One of the most agronomically important forage grasses, *Festuca pratensis* Huds. is treated as a model plant to study the molecular mechanisms associated with tolerance to winter stresses, including frost. However, the precise mapping of the genes governing stress tolerance in this species is difficult as its karyotype remains unrecognized. Only two *F*. *pratensis* chromosomes with 35S and 5S rDNA sequences can be easily identified, but its remaining chromosomes have not been distinguished to date. Here, two libraries derived from *F*. *pratensis* nuclear DNA with various contents of repetitive DNA sequences were used as sources of molecular probes for fluorescent *in situ* hybridisation (FISH), a BAC library and a library representing sequences most frequently present in the *F*. *pratensis* genome. Using FISH, six groups of DNA sequences were revealed in chromosomes on the basis of their signal position, including dispersed-like sequences, chromosome painting-like sequences, centromeric-like sequences, knob-like sequences, a group without hybridization signals, and single locus-like sequences. The last group was exploited to develop cytogenetic maps of diploid and tetraploid *F*. *pratensis*, which are presented here for the first time and provide a remarkable progress in karyotype characterization.

## Introduction

Grasses (Poaceae) constitute a large family of monocotyledonous plants. This family encompasses cereals, turf and pasture grasses, and others such as bamboos or sugarcane. The area of grassland is estimated to be two times higher than the area of cropland. However, the genetic/genomic resources for grassland species are still limited [1, 2]. The most important forage grasses used in agriculture are species derived from the *Festuca-Lolium* complex, i.e. *Festuca pratensis* Huds. (meadow fescue), *F*. *arundinacea* Schreb. (tall fescue), *Lolium multiflorum* Lam. (Italian ryegrass), and *L*. *perenne* L. (perennial ryegrass). *Festuca* species are characterized by high tolerance to abiotic and biotic environmental stresses. On the contrary, *Lolium* species possess higher forage quality and productivity in favourable conditions but a significantly lower performance under stresses. The species of these two genera are closely related and their desirable complementary agronomic traits can be combined in intergeneric hybrids and their introgression derivatives [3–6]. However, the mapping of the genes governing these important traits in particular species and in their intergeneric hybrids is difficult as the majority of chromosomes within *Festuca* and *Lolium* species cannot be unambiguously recognized.

Cytogenetics has proved to be an important tool to determine evolutionary and phylogenetic relationships between various plant groups, as well as to uncover the genome structure at the chromosomal level [7–9]. Fluorescence *in situ* hybridization (FISH) is one of the most important and useful methods that provide chromosome landmarks. FISH in combination with large-insert BAC-based clones restricted to single locus sequences have been efficiently used in karyotype analyses of such plant species as *Brachypodium pinnatum* [10], *Daucus carota* [11] and *Phaseolus microcarpus* [12]. Sequencing revealed that in plant genomes repetitive sequences constitute most of the DNA and represent up to 85% of the genome size [13–16]. This high content of repetitive sequences often causes trouble in the identification of single locus sequences that enable the recognition of individual chromosomes. However, this type of sequences, e.g. the *Afa*-family in the *Hordeum vulgare* karyotype, could be useful in chromosome identification [17].

The *Festuca* genus includes more than 500 species and is thought to be the most numerous and complex genus of Poaceae [18]. The ploidy level of *Festuca* species ranges from diploid (2n = 2x = 14) up to dodecaploid (2n = 12x = 84) [19]. The haploid genome size in *Festuca* ranges from 1.638 Gbp to 12.553 Gbp (per 1C DNA) [20]. The sequencing of the 4F chromosome of *F*. *pratensis* revealed that the most abundant repeats (about 30%) constitute Ty3-*gypsy*-like elements [21].

Some chromosomes of *Festuca* can be recognized by 5S and 35S rDNA sequences [22–24]. In the *F*. *pratensis* karyotype the chromosome no. 2 (2F) with a secondary constriction (NOR) and the chromosome no. 3 (3F) with 5S rDNA loci are well-recognized (chromosome nomenclature according to Thomas et al. [22]). However, although this species is treated as a model plant within the group of *Festuca*-*Lolium* forage grasses to recognize the molecular basis of mechanisms involved in tolerance to low temperature, including frost [5, 25], its remaining chromosomes have not been well-characterized to date [26].

In this paper, two libraries derived from *F*. *pratensis* nuclear DNA with various contents of repetitive DNA sequences, a BAC library and library representing sequences most frequently present in the *F*. *pratensis* genome, were used in a cytogenetic approach. We hypothesized that these two libraries could be an excellent source of molecular probes to be applied for the FISH procedure aiming at the identification of *F*. *pratensis* chromosomes and their particular arms. Thus, the main goal of the present study was to characterize the karyotype of diploid and tetraploid *F*. *pratensis* cultivars using BAC clones and probes enriched with sequence repeats, and finally to create the first detailed cytogenetic maps of this species.

## Materials and methods

### Plant material

The plant materials consisted of diploid (2n = 2x = 14) *F*. *pratensis* cv. Skra and autotetraploid (2n = 4x = 28) cv. Westa. Diploid plants with additional B chromosomes (2n = 2x = 14+2B) and plants with a chromosome carrying an additional 5S rDNA locus were also included in the analysis. Additionally, *F*. *pratensis* × *L*. *perenne* (2n = 4x = 28) intergeneric hybrids were used.

### Construction of a *F*. *pratensis* genomic DNA library

To study *F*. *pratensis* genome organization, a genomic library from *F*. *pratensis* nuclear DNA in a cloning vector was prepared. The DNA library, representing sequences most frequently present in the *F*. *pratensis* genome, was constructed according to the procedure of Nunome et al. [27]. Genomic DNA was isolated from *ca*. 100 mg of powdered leaves using the GeneJET Plant Genomic DNA Purification Kit (Thermo Scientific). The quality and quantity of DNA were analyzed in a 1% agarose gel and using a NanoDrop photometer (Thermo Scientific). Total genomic DNA was digested in a volume of 100 μl with 25 units of the restriction endonucleases *Hin*dIII (Promega) for 17 h at 37°C. The enzyme was inactivated by heating for 10 min at 65°C. Digested DNA was purified by extractions with phenol/chloroform and precipitation with 100% ethanol, and then subcloned into the *Hin*dIII cloning sites of the pBluescript KS(+) vector. To reduce the background of “empty” clones, digested plasmid DNA was dephosphorylated using alkaline phosphatase (calf intestinal; Promega). Ligation was performed in a total volume of 10 μl using 3 units of T4 DNA ligase (Promega) and the molar ratio of insert to vector was 3:1. Library construction and screening were performed directly by transformation of a ligate into competent cells of XL1Blue MRF’ *E*. *coli*. Putative positive clones were screened on selective LB medium containing ampicillin at 100 μg.ml^-1^, and subjected to isolation of plasmid DNA using GeneJet Plasmid Miniprep Kits (Thermo Scientific). The lengths of 469 clones were estimated by separation in 1% agarose gels.

### Fluorescent *in situ* hybridization

#### Chromosome preparation

Metaphase accumulation and fixation procedures, as well as the preparation of chromosome slides for *in situ* hybridization, were carried out by the enzymatic squash method according to the protocol described by Książczyk et al. [24].

#### Probes

For the experiments presented here, BAC clones and a DNA library, representing sequences most frequently present in the *F*. *pratensis* genome, were used. From the BAC library moderate- and low-repetitive clones were selected on the basis of previously performed dot blot analysis. From the newly created library clones were chosen on the basis of their molecular size. In total, 131 BAC-based probes and 61 molecular probes enriched with sequence repeats were physically verified. To identify some chromosomes within the karyotype, rDNA sequences were used. The 5S rDNA probe was derived from *Triticum aestivum* clone pTa794 [28] and the 35S rDNA probe was generated from the coding region of 26S rDNA of *Arabidopsis thaliana* [29].

Preparation of probes involved (*i*) isolation of plasmid DNA using the standard alkaline extraction procedure as described by Farrar and Donnison [30]; (*ii*) nick-translation labelling of 35S rDNA and the clones derived from the two *F*. *pratensis* libraries using digoxigenin-11-dUTP (Sigma-Aldrich), tetramethyl-5-dUTP-rhodamine (Sigma-Aldrich) or Atto647N-dUTP (Jena BioScience); (*iii*) PCR labelling of 5S rDNA with tetramethyl-5-dUTP-rhodamine (Sigma-Aldrich).

#### Hybridization

FISH experiments were done according to Książczyk et al. [31] with minor modifications. Briefly, the hybridization mixture contained 100 ng of each probe in the presence of 50% formamide, 2 × SSC and 10% dextran sulphate. All the clones were hybridized separately, one clone per one chromosome slide. Chromosomal DNA in hybridization mixture was denatured at 80°C for 2 min and allowed to hybridize overnight at 37°C. For the detection of the hybridization signals of probes labelled with digoxigenin, anti-digoxigenin conjugated with fluorescein isothiocyanate (FITC) was used.

Moreover, the location of selected clones was checked in relation to each other. The reprobing was conducted by first removing particular probes before the next round of hybridization with another probe, according to the procedure described by Heslop-Harrison [32]. The procedure involved washing in 4 × SSC with 0.2% Tween 20 (Sigma-Aldrich) three times per 10 min, and in 2 × SSC twice per 10 min. Then, the slides were dehydrated in an ethanol series at room temperature. The sets of probes were subsequently applied on each slide followed by the application of rDNA probes. In total three rounds of hybridization with the clones, and the fourth one with rDNA sequences, were performed.

All the steps in the genomic *in situ* hybridization (GISH) protocol were performed according to the Kosmala et al. [5] procedure with minor modifications. In GISH, the total genomic DNA of *L*. *perenne* was used as a probe and labelled with digoxigenin using a DIG-Nick Translation Kit according to the manufacturer’s procedure (Sigma-Aldrich). Genomic DNA of *F*. *pratensis* was used as blocking DNA after being sheared by boiling for 30 min.

All the images were acquired using an Olympus XM10 CCD camera attached to an Olympus BX 61 automatic epifluorescence microscope. Image processing was carried out using Olympus Cell-F imaging software (ver. 3.1; Olympus Soft Imaging Solutions GmbH, Germany) and Micrographx Picture Publisher software (ver. 10; Corel Corporation, Canada). For the cytogenetic maps which were created, the distributions of hybridization signals in the chromosomes is shown in relative lengths determined as means of ten measurements.

## Results

### A DNA library representing sequences most frequently present in the *F*. *pratensis* genome

DNA digested by *Hin*dIII was used for the construction of a genomic DNA library. After the isolation of inserted DNA, the clones were divided into groups according to their molecular size. A classification of clones is shown in Table 1. The most numerous class that was physically mapped in diploid *F*. *pratensis* chromosomes comprises sequences with a length of 10 000 bp and more. However, some clones from other groups were also verified to give signals on chromosomes. The clones with length smaller than 3 500 bp were not cytogenetically mapped due to the limited resolution of metaphase chromosomes in the FISH technique.

**Table 1:** Classification of selected molecular probes enriched with DNA sequence repeats according to their size. Clones which were not cytogenetically analysed are indicated by NA.

Size of clones [bp]	>10.000	10 000	8 000	6 000	5 000	4 000	3 500	3 200	3 000	2 500	2 300	2 000
**Number of clones in this group**	109	22	22	20	7	12	22	32	46	53	85	39
**Number of clones cytogenetically verified**	44	NA	2	4	6	NA	5	NA	NA	NA	NA	NA

### Chromosome nomenclature

For *Festuca* and *Lolium* species, two types of chromosome nomenclature exist according to the numbering systems of Thomas et al. [33] and of Triticeae [34]. Although in this work the nomenclature of Thomas et al. [33] was used, in the present study we propose a new nomenclature for the karyograms and cytogenetic maps which we created. This new nomenclature was developed due to the unambiguous identification of the first to seventh chromosome pairs identified by Thomas et al. [33]. We named chromosomes with letters from A to G. Chromosomes 2F and 3F (according to Thomas et al. [33]) correspond to our C and D chromosomes, respectively. Furthermore, a chromosome with an additional 5S rDNA locus was named chromosome E2. Chromosome E2 and chromosome E without rDNA sequences are presented separately on the karyograms.

### Chromosome mapping

In order to achieve a high genome coverage for the unmapped *F*. *pratensis* chromosomes, 131 BAC-based probes and 61 molecular probes enriched with sequence repeats were examined cytogenetically. Six groups of hybridization patterns were observed: (1) dispersed-like sequences, (2) chromosome painting-like sequences, (3) centromeric-like sequences, (4) knob-like sequences, (5) a group without hybridization signals, and (6) single locus-like sequences (Table 2). The dispersed-like and chromosome painting-like sequences should be classified into the same group, because both represent highly repetitive sequences specific to species or genomes. However, in this paper they were divided into two separate groups on the basis of their distribution in chromosomes and potential usefulness for more detailed analysis of chromosome identification of *Festuca*-*Lolium* hybrids.

**Table 2:** Types and numbers of hybridization patterns after FISH with BAC-based probes and molecular probes enriched with sequence repeats in the *F*. *pratensis* genome.

***F*. *pratensis* BAC library (131 clones in total)**						
Dispersed-like sequences		Chromosome painting-like sequences	Centromeric-like sequences	Knob-like sequences	Lack of signals	Single locus-like sequences
67		5	6	13	31	9
**DNA library representing sequences most frequently present in *F*. *pratensis* genome (61 clones in total)**						
Dispersed-like sequences		Chromosome painting-like sequences	Centromeric-like sequences	Knob-like sequences	Lack of signals	Single locus-like sequences
18		16	10	4	11	2
**Total:**	**85**	**21**	**16**	**17**	**42**	**11**

**Fig 1:**
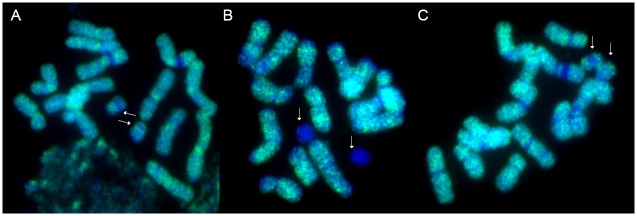
Chromosome painting-like sequences: Distribution in supernumerary B chromosomes of *F*. *pratensis*. A) distribution of clone 185; B) distribution of clone 183; C) distribution of clone 212. All the clones were labelled with FITC (green), and chromosomes were counterstained with DAPI (blue). White arrows indicate the supernumerary B chromosomes.

**Fig 2:**
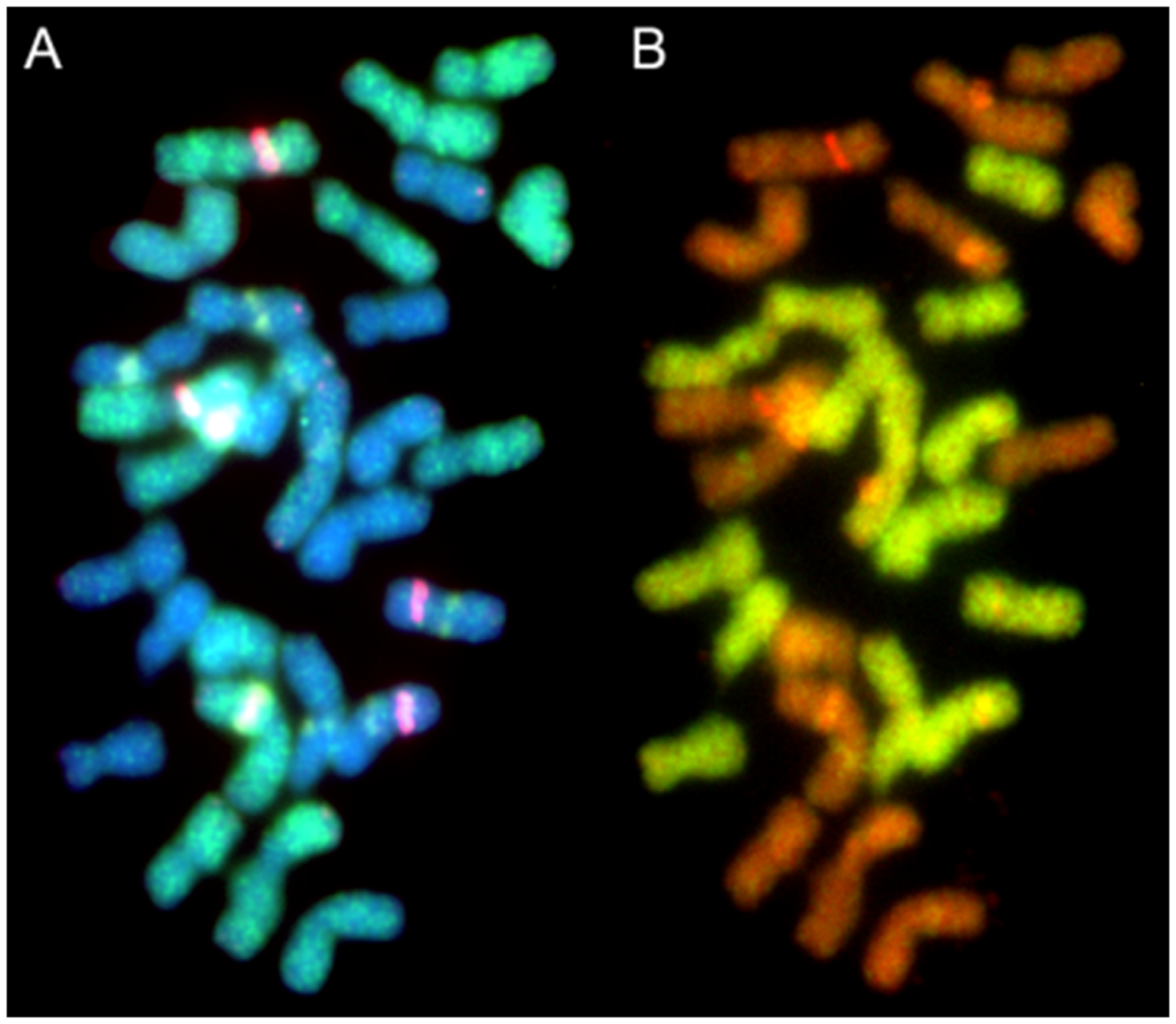
Genome painting-like sequence specific for *F*. *pratensis* chromosomes in a *F*. *pratensis* × *L*. *perenne* hybrid. A) Distribution of clone L16 Fp04 (green), 5S rDNA (red) and 35S rDNA (green); chromosomes were counterstained with DAPI (blue); B) GISH with the total genomic DNA of *L*. *perenne* (green); chromosomes were counterstained with propidium iodide (orange).

The highest number of sequences within a group constituted of dispersed signals along both arms of the chromosomes (group no. 1; 85 clones) was found. Within the chromosome painting class (group no. 2; 21 clones) it was noticed that only one clone (393) mapped to the whole chromosomes, while the remaining chromosome painting-like sequences hybridized to the entirety of chromosomes excluding segments of the 35S rDNA loci (NOR) (Fig 1A), the NOR and centromeric regions (Fig 1B), as well as to chromosomes without centromeric regions (Fig 1C). Moreover, hybridization of clone 183 to the genotype with additional B chromosomes revealed that this sequence is specific for the A chromosome set (Fig 1B). The hybridization pattern of the remaining chromosome painting-like sequences resulted in signals that were present in supernumerary B chromosomes without pericentromeric (Fig 1A) or centromeric regions (Fig 1C). Within chromosome painting-like sequences, one species-specific clone (L16 Fp04) was identified; hybridization of this clone to chromosomes of a *F*. *pratensis* × *L*. *perenne* hybrid revealed signals restricted to the *F*. *pratensis* chromosomes. In Fig 2 the same metaphase plate after FISH with clone L16 Fp04 (Fig 2A) is compared with results obtained from GISH using total genomic DNA of *L*. *perenne* as a probe (Fig 2B).

**Fig 3:**
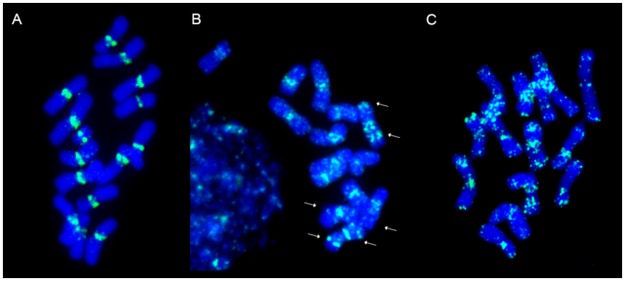
FISH patterns of the centromeric group of clones. A) Clone 639 restricted to centromeric regions; B) Clone 433 specific for centromeric regions and some interstitial regions (white arrows); C) Clone 171 specific for centromeric and telomeric regions. All clones were labelled with FITC (green); chromosomes were counterstained with DAPI (blue).

**Fig 4:**
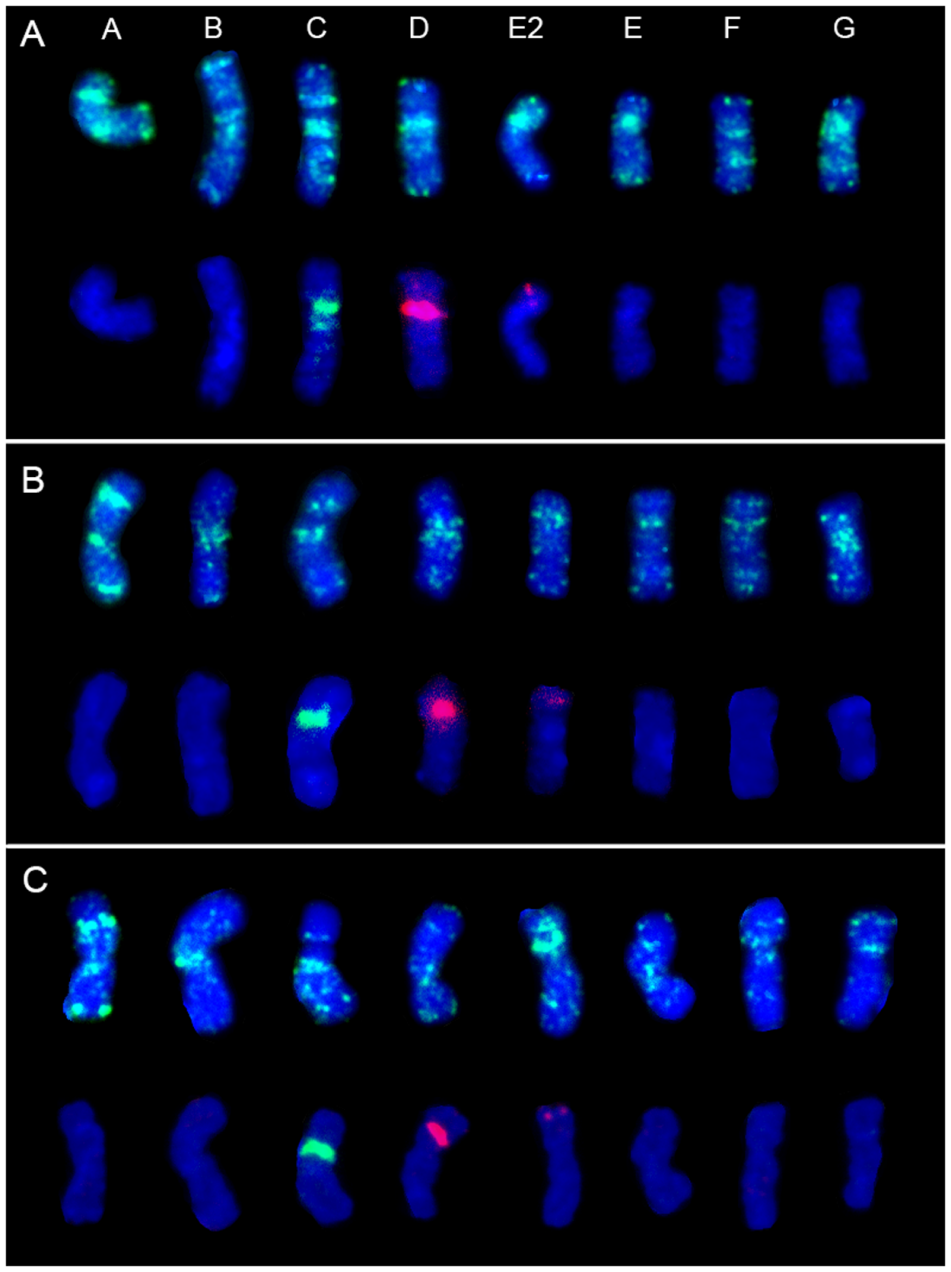
FISH patterns on mitotic chromosomes of diploid *F*. *pratensis* using: A) clone 595, B) clone 616, C) clone G18 Fp01. In panels A, B and C, in the upper rows the distribution of BAC clones is presented; all the clones were labelled with FITC (green). In the lower rows of each panel the 5S (red) and 35S (green) rDNA loci are presented. Chromosome E2 with an additional 5S rDNA locus and chromosome E without rDNA are presented separately. Chromosomes were counterstained with DAPI (blue).

The group no. 3 encompasses sequences specific for centromeric regions of chromosomes. Within this group sixteen clones were identified, including clones with signals restricted to centromeric regions (Fig 3A), clones labelling (peri)centromeric regions, and some interstitial positions (Fig 3B), as well as one clone (171) which was mapped in both (peri)centromeric and telomeric regions (Fig 3C). Hybridization patterns of the selected clones specific for centomeric regions with interstitial signals within chromosomes might be useful to facilitate chromosome identification (Fig 4). In Fig 4A, 4B and 4C the physical distribution of three sequences is shown (clone 595, 616 and G18 Fp01).

Among the clones analysed, knob-like sequences (group no. 4; 17 clones) were also identified. These sequences hybridized to specific and random blocks on chromosomes. The vast majority of them were mapped to segments located in all chromosomes. Clone 266 revealed a signal in telomeric and subtelomeric regions of all of the chromosomes. Similarly, several clones classified to this group labelled interstitial regions in both arms of all of the chromosomes (S1 Fig). Clones which revealed no hybridization signals were also identified in both libraries (group no. 5; 42 clones). The majority of unmapped clones was characterized by a low physical size of the inserted DNA in the library representing sequences most frequently present in the *F*. *pratensis* genome.

**Fig 5:**
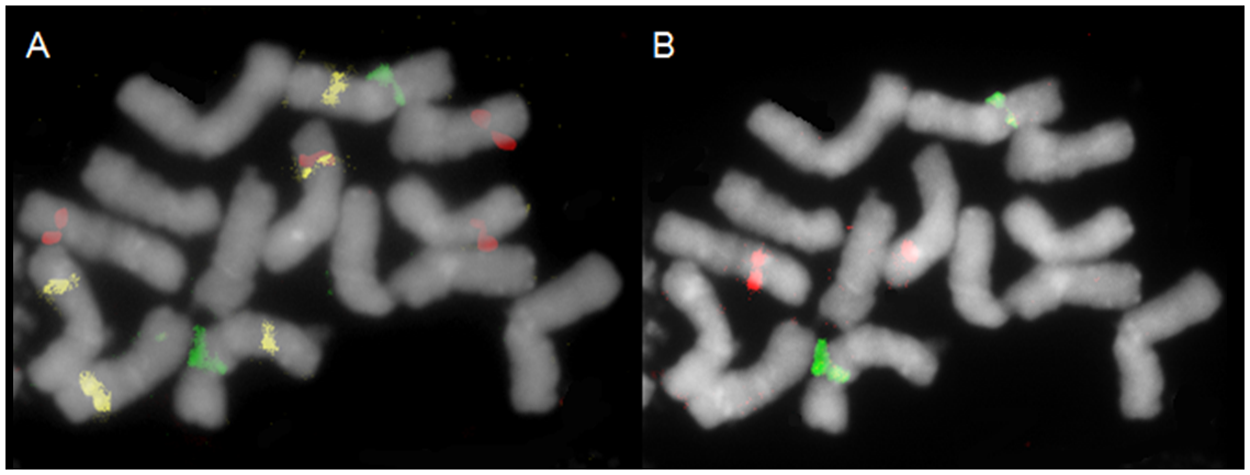
The distribution of selected single locus clones in *F*. *pratensis* chromosomes. A) E5 Fp04 (green), K11 Fp04 (red), A21 Fp02 (yellow). B) The position of clones compared to the location of 35S rDNA (green) and 5S rDNA (red) after reprobing.

The last group of hybridization patterns consisted of clones apparently mapped to a single locus (group no. 6; 11 clones), of which the majority were derived from the BAC library. However, two single locus clones (clones 228 and 324) were also identified in the library representing sequences most frequently present in the *F*. *pratensis* genome. Some of these showed clear signals in an individual pair of chromosomes (Fig 5), but for some of them weak dispersed signals in the remaining chromosomes were also observed (data not shown). In Fig 5A the physical location of selected single locus clones is presented. The position of one of these clones (E5 Fp04) co-localized with 35S rDNA (Fig 5B). Clone K11 Fp04 was mapped to two pairs of chromosomes, including one unrecognized pair and one bearing 5S rDNA (chromosome pair D). However, in chromosome D this clone hybridized to arms without rDNA loci. The last clone (A21 Fp02) was mapped to five chromosomes: two C chromosomes in the arm without 35S rDNA loci, one pair without recognized landmarks, and only one D chromosome in the arm without 5S rDNA (Fig 5A).

**Fig 6:**
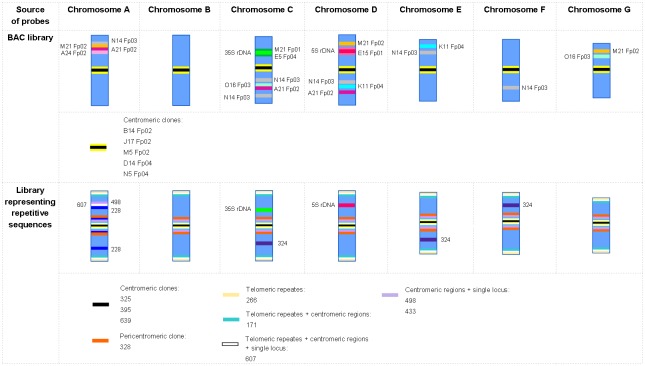
The cytogenetic map of diploid *F*. *pratensis*. The ideograms show the relative chromosome length, the position of centromeres, the distribution of rDNA loci, and the position of mapped clones with their origin. The positions of hybridization signals in the chromosomes are shown as relative lengths.

The distribution of selected clones is presented in Table 3. The results of FISH experiments are summarized on the ideograms of *F*. *pratensis* chromosomes (Fig 6). To verify the position of clones in unrecognized chromosomes, experiments using different sets of probes on the same chromosome slides were performed, and rDNA-FISH was also carried out on these slides to discriminate rDNA-bearing chromosomes.

**Table 3:** Distributions of selected clones in diploid and tetraploid forms of *F*. *pratensis*. Positions of clones in rDNA-bearing chromosomes were assigned by reference to rDNA sequences; (M+) is the arm with an rDNA marker and (M-) the arm without an rDNA marker.

Name of clone	Distribution in diploid *F*. *pratensis*	Distribution in tetraploid *F*. *pratensis*
E15 Fp01	D chromosomes (M+)–colocalization with 5S rDNA sequence	centromeric-like sequences, D chromosomes (M+), four unrecognized chromosomes
G18 Fp01	diversified pattern across all chromosomes (see Fig 4C)	centromeric-like sequences
M21 Fp01	C chromosomes (M+)–colocalization with 35S rDNA sequence	C chromosomes (M+)–colocalization with 35S rDNA sequence
A21 Fp02	C chromosomes (M-), one D chromosome (M-), two unrecognized chromosomes	C chromosomes (M-), two unrecognized chromosomes
A24 Fp02	C chromosomes (M-)	lack of signals
M21 Fp02	D chromosomes (M+), four unrecognized chromosomes	C chromosomes (only in one pair M+), four unrecognized chromosomes
N14 Fp03	C chromosomes (M-), D chromosomes (M-), eight unrecognized chromosomes	centromeric-like sequences
O16 Fp03	C chromosomes (M-), two unrecognized chromosomes	lack of signals
E5 Fp04	C chromosomes (M+)–colocalization with 35S rDNA sequence	C chromosomes (M+)–colocalization with 35S rDNA sequence
K11 Fp04	D chromosomes (M-), two unrecognized chromosomes	lack of signals
228	two unrecognized chromosomes	four unrecognized chromosomes
324	C chromosomes (M-), four unrecognized chromosomes	telomeric-like sequences, C chromosomes (in one pair M+, in second M-), four unrecognized chromosomes
433	centromeric-like sequences, two unrecognized chromosomes	centromeric-like sequences, four unrecognized chromosomes
498	centromeric-like sequences, two unrecognized chromosomes	centromeric-like sequences, four unrecognized chromosomes
595	diversified pattern across all chromosomes (see Fig 4A)	centromeric-like sequences, telomeric-like sequences, four unrecognized chromosomes
607	centromeric-like sequences, telomeric-like sequences, two unrecognized chromosomes	centromeric-like sequences, four unrecongized chromosomes
616	diversified pattern across all chromosomes (see Fig 4B)	centromeric-like sequences, C chromosomes (M+)

**Fig 7:**
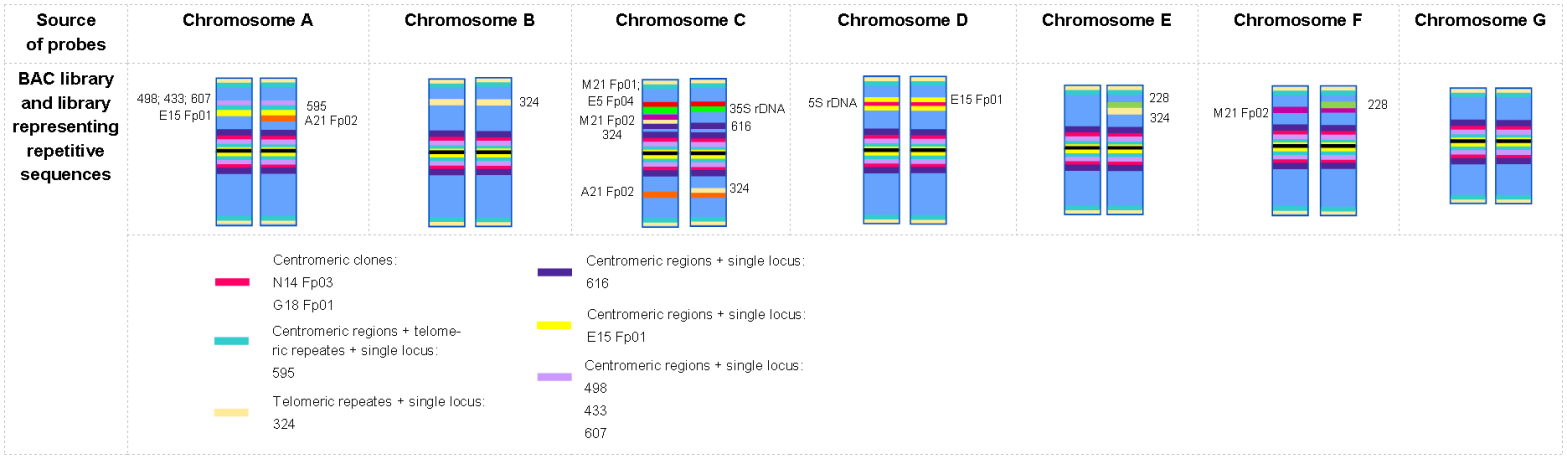
The ideogram of autotetraploid *F*. *pratensis* based on both DNA libraries. The ideograms show the relative chromosome length, the position of centromeres, the distribution of rDNA loci, and the positions of the mapped clones. The positions of hybridization signals in the chromosomes are given as relative lengths.

A pool of 17 clones was used to build a cytogenetic map of diploid *F*. *pratensis* and to examine similarities and differences between diploid and tetraploid form of this species with respect to their hybridization patterns (Table 3). In the tetraploid form, the clones 433 and 498 were mapped to almost the same positions, centromeric regions and the four largest metacentric A chromosomes. In the diploid form, signals of these clones were observed in centromeric regions and the two largest chromosomes. A similar pattern for clone 607 was also noticed, except for the telomeric-like sequences that were mapped on chromosomes of the diploid form only. Clone 228 was mapped in two and four unrecognized chromosome in diploid and tetraploid *F*. *pratensis*, respectively. Clones M21 Fp01 and E5 Fp04, specific for the 35S rDNA position in diploid *F*. *pratensis*, were also mapped to the 35S rDNA loci in the tetraploid form. Surprisingly, clone E15 Fp01, which was colocalized with 5S rDNA in the diploid form, was mapped in centromeric regions beyond the 5S rDNA loci in the tetraploid form. Sequence A21 Fp02 hybridized to C chromosomes, two unrecognized chromosomes, and to one D chromosome in diploids. In the tetraploid form this sequence mapped to C chromosomes and two unrecognized chromosomes. An intriguing position of clone 324 was observed in C chromosomes of tetraploids, where in one pair of chromosomes the sequence was visible in an arm without 35S rDNA loci, while in the second pair of chromosomes it was in an arm with 35S rDNA. Furthermore, in the diploid form this clone mapped to four unique positions, while in the tetraploid form four single locus signals plus additional signals in telomeric/subtelomeric regions were observed. The signals of M21 Fp02 in diploid *F*. *pratensis* were observed in D chromosomes and in four unrecognized chromosomes, but in the tetraploid form this sequence was visible in one pair of C chromosomes and four unrecognized chromosomes. Moreover, signals of clone N14 Fp03, which was mapped to selected chromosomes in the diploid form, were restricted to centromeric regions in the tetraploid form. Centromeric regions in the tetraploid form were also marked by two other clones (595 and 616). Additionally, for clone 595 four signals in unrecognized chromosomes were noticed, as well as telomeric-like signals in all chromosomes. For clone 616 two signals were visible in two C chromosomes, in the arms bearing 35S rDNA sequences. Three clones (A24 Fp02, O16 Fp03, K11 Fp04) revealed no signals in tetraploid *F*. *pratensis*. The schematic position of all mapped clones in tetraploid *F*. *pratensis* is shown in the ideogram (Fig 7).

## Discussion

The BAC-FISH technique has not been widely used in *Festuca* and *Lolium* species. Until now no cytogenetic or physical map has been published for *F*. *pratensis* or for other species from the *Festuca*-*Lolium* complex. The available cytogenetic data about the karyotype structure of *F*. *pratensis* was restricted to the morphology of chromosomes and the identification of two pairs of chromosomes using rDNA-FISH [22, 24]. In this study, our knowledge of genome organization of this species was extended. A novel source of genomic data, a library representing sequences most frequently present in the *F*. *pratensis* genome, has been developed and mapped in chromosomes. Moreover, clones from a BAC library containing a relatively high proportion of unique sequences were also used to analyze the chromosomes.

Although little is known about the structure of *Festuca* chromosomes, except for rDNA distribution [22, 24], an effort has been invested in the last years to recognize these chromosomes. Kopecký et al. [26] showed the result of mapping BAC 1G18 in individual chromosomes of *F*. *pratensis* introgressed into *L*. *multiflorum*. This BAC clone probably contains a high amount of repetitive DNA, because signals were observed mostly in centromeric regions. Almost the same results were obtained in our experiments for C and D chromosomes (Fig 4C), but some discrepancies between both analyses were also detected including a higher number of signals within all of the chromosomes in the present study, especially in the interstitial locations. The occurrence of minor differences could be related to different types of plant material used in both studies (a cultivar of *F*. *pratensis* and an introgression form of *L*. *multiflorum* with introgressed individual *F*. *pratensis* chromosomes) or to different conditions of post hybridization washes.

The availability of BAC-based chromosome-specific landmarks has enabled, for example, a more detailed analysis of the karyotype of *Brachypodium* species, including insight into the karyotype evolution [10, 35]. In our study, a large pool of clones provided a good possibility to characterize the *F*. *pratensis* karyotype. The application of all of the identified single locus clones in combination with several repetitive sequences allowed to identify individual chromosomes and to develop cytogenetic maps of *F*. *pratensis* (Fig 6; Table 3) using a total of 28 out of 192 clones. The highest number of landmarks was found for one of the largest metacentric chromosome. Nevertheless, for one chromosome (named with letter B) single locus sequences were not found. In species with small genomes, such as *Arabidopsis thaliana* [36], *Brachypodium distachyon* [37] and *Lotus japonicus* [38], cytogenetic maps have been constructed using BAC clones as probes, enabling the efficacious integration of cytogenetic, physical and genetic maps. Integrated physical and genetic maps could be helpful in studies of genome organization and evolution, as well as for anchoring of whole genome shotgun sequences [39, 40].

Sequences derived from genomic DNA libraries can be used for chromosome painting. Interestingly, we observed only one clone (393) which painted the entire karyotype. Several other clones produced a signal over all the chromosomes with the exception of 35S rDNA loci and/or centromeric regions. Centromeric regions are mainly composed of tandem repeats and retrotransposons [41, 42], and centromere-specific sequences including *crwydryn* elements in rye [43]), *cereba* in barley [41], and *CRM1* and *CRM4* in maize [44] have been identified. Such elements may also exist in the *F*. *pratensis* genome, but were absent in the clones producing signals over all chromosomes except the centromeric regions.

Chromosome painting clones enable ancestral genome recognition in allopolyploid species. Zhang et al. [45] identified sequences that distinguish the three genomes of *Triticum aestivum*, and in *Cucumis metuliferus* a species-specific repetitive sequence (pMetSat) has been identified by Yagi et al. [46]. In our study, we observed a hybridization signal of the chromosome-like painting clone L16 Fp04 only in *F*. *pratensis* chromosomes in *F*. *pratensis* × *L*. *perenne* hybrids, indicating a species-specificity of this sequence. Moreover, we identified clone 183, which is specific for the A chromosome complement, because signals in additional B chromosomes did not occur. Houben et al. [47] found that supernumerary B chromosomes of rye incorporate large amounts of B-specific repeats as well as insertions from cytoplasmic organellar DNA. This might explain the lack of signals in additional B chromosomes in the *F*. *pratensis* karyotype (Fig 1B). On the other hand, most of the clones produced visible signals in both A and B sets (Fig 1A and 1C) supporting the hypothesis that supernumerary B chromosomes originated as a by-product of A chromosome evolution [48]. Thus, some sequences are probably common to both sets of chromosomes.

The physical mapping of selected clones in diploid and tetraploid *F*. *pratensis* revealed different hybridization patterns, e.g. clone N14 Fp03, which was mapped to selected chromosomes of the diploid form, resulted in centromeric-like signals in tetraploid forms (Fig 7, Table 3). This is consistent with the hypothesis that a polyploid is not simply the sum of the diploid parents and that many changes including chromosome rearrangements appear after polyploidization [49]. For example, elimination of low-copy sequences derived from diploid progenitors has been observed in hexaploid bread wheat [50]. Probably, sequences A24 Fp02, O16 Fp03 and K11 Fp04, which gave single locus signals in the diploid form, were eliminated during the evolution of tetraploid forms (Fig 7, Table 3). Large scale re-organization including inversions, duplications and translocations has been evidenced in many plant polyploids [51, 52]. Similarly, we found chromosome rearrangements in autopolyploid plants of *F*. *pratensis*; the clone 324 was mapped in two pairs of C chromosomes in tetraploids but the distribution of signal differs and one pair carries a signal in the arms with 35S rDNA loci, while in the second pair this sequence hybridized to the arms without 35S rDNA (Fig 7, Table 3). What is more, changes in the structure of D chromosome between both ploidy levels were identified; hybridization of clone A21 Fp02 resulted in signals in only one chromosome D and this clone did not give signals in this chromosome pair at the tetraploid level (Table 3). Similarly we observed hybridization signals for M21 Fp02 only in the D chromosomes in diploids but no signals in the tetraploid form. However, in the autotetraploid form this sequence was mapped in another rDNA-bearing chromosome (chromosome C) but in only one out of two chromosome pairs. Chromosomes C and D in the karyotype of *F*. *pratensis* bear rDNA loci, and it was previously reported that rDNA sequences can be highly polymorphic [24]. However, it is worthwhile to mention that most of the clones appeared in the expected numbers and positions in autotetraploid plants.

This research demonstrates the potential of using repetitive and unique types of sequences derived from BAC and genomic DNA libraries for the development of cytogenetic markers. These libraries allowed more detailed characterization of the chromosome complement of *F*. *pratensis*. A large group of clones constitutes sequences with a high amount of repetitive DNA, restricted to centromeric/pericentromeric and telomeric regions. Furthermore, the comparative mapping between *Festuca* and *Lolium* species also confirmed that these genomes consist of large number of repetitive sequences [21, 53].

Our results could be a helpful reference and a good starting point for further research to identify the particular chromosomes of this species within the chromosomal set in intergeneric hybrids of *F*. *pratensis* with *Lolium* species. Moreover, this approach could be simultaneously associated with physical mapping of chromosomal regions carrying genes governing tolerance to environmental stresses, including low temperature, in *F*. *pratensis* and its intergeneric *Festuca* × *Lolium* hybrids.

## Supporting information

S1 Fig The distribution of selected knob-like sequences in *F*. *pratensis* (2n = 2x = 14) chromosomes. The physical distribution of: A) clone 282 (green); B) clone N12 Fp04 (green) and C) clone N16 Fp04 (green). On the same metaphase plates 35S rDNA (green) and 5S rDNA (red) sequences were mapped. Chromosomes were counterstained with DAPI (blue).
(TIF)
