# Supplementary figures and images for: Exploiting repetitive sequences and BAC clones in Festuca pratensis karyotyping
Source: PLoS One. 2017 Jun 7;12(6):e0179043. doi: 10.1371/journal.pone.0179043 (PMC5462415; doi:10.1371/journal.pone.0179043)

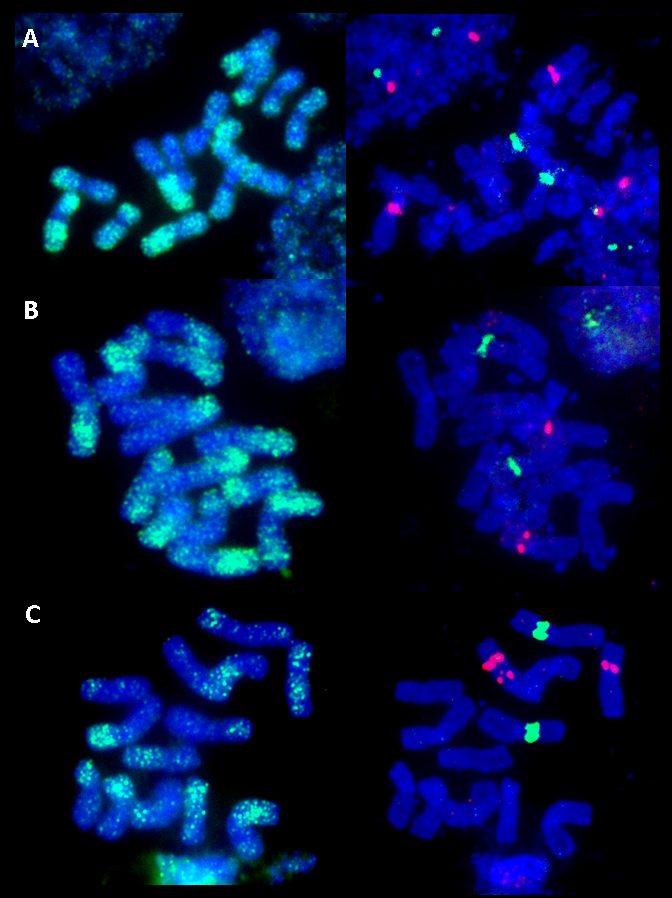

Supplement: S1 Fig — The physical distribution of: A) clone 282 (green); B) clone N12 Fp04 (green) and C) clone N16 Fp04 (green). On the same metaphase plates 35S rDNA (green) and 5S rDNA (red) sequences were mapped. Chromosomes were counterstained with DAPI (blue). (TIF) [file pone.0179043.s001.tif]
